# Influence of Admixture Source on Fresh Properties of Self-Consolidating Concrete

**DOI:** 10.3390/ma17133215

**Published:** 2024-07-01

**Authors:** Nader Ghafoori, Aderemi Gbadamosi, Hamidou Diawara, Ariful Hasnat

**Affiliations:** Department of Civil and Environmental Engineering and Construction, University of Nevada, 4505 Maryland Parkway, P.O. Box 454015, Las Vegas, NV 89154-4015, USA; nader.ghafoori@unlv.edu (N.G.); hdiawara@gmail.com (H.D.); hasnat@unlv.edu (A.H.)

**Keywords:** self-consolidating concrete, polycarboxylate-based high-range water-reducing admixture, viscosity-modifying admixture, flowability, viscosity, stability, passing ability, filling ability

## Abstract

The study presented herein was intended to (1) compare the optimum (minimum) dosage requirements of four different sources of polycarboxylate-based high-range water-reducing admixtures (HRWRAs) and viscosity-modifying admixtures (VMAs) in attaining slump flows of 508 mm, 635 mm, and 711 mm, and a visual stability index (VSI) of 0 (highly stable concrete) or 1 (stable concrete), and (2) assess the flowability/viscosity, stability, passing ability, and filling ability of the resulting self-consolidating concretes. The test results showed that the optimum dosage requirements to obtain a uniform slump flow and visual stability index varied among the four selected admixture sources. The required dosage amount for HRWRAs was highest for the polycarboxylate-ester (PCE) type and lowest for the polycarboxylate-acid (PCA) type. Acceptable flowability plastic viscosity dynamic and static stability, passing ability, and filling ability of self-consolidating concrete can be achieved with the proper dosing of the four studied admixture sources.

## 1. Introduction

Self-consolidating concrete (SCC) mixtures are a unique type of concrete mix with adequate workability without requiring any kind of internal or external compaction. They can flow under their own weight, completely fill every corner of the formwork, and achieve full compaction even in the presence of crowded reinforcement [1,2,3,4,5,6]. When compared to vibratory-placed concrete, SCCs offer better consolidation around reinforcements, minimize disturbances because vibration is eliminated, enhance the surface profile, and improve working conditions and safety [7,8,9,10,11,12]. SCCs were developed in the late 1980s by Prof. Okamura and his coworkers at the University of Tokyo, Japan [12,13,14]. The main objective of contemporary SCCs is to provide a mix with a low yield and sufficient viscosity to spread freely without vibration or compacting effort [15].

Recent developments in concrete technology have made it possible to customize chemical admixtures to meet certain construction requirements and produce workable concrete without losing strength [16]. Due to their greater benefits over traditional concrete, SCCs have been utilized by the construction industry for the past two decades and are one of the products that have benefited from the advancements in concrete technology. High-range water-reducing admixtures (HRWRAs) or superplasticizers (SP) and viscosity-modifying admixtures (VMA) are used to adjust the workability and viscosity of SCCs. HRWRAs create the necessary flowability through the adsorption of cement particles and by inducing an electrical charge that limits the formation of cement flocs [17]. Presently, there are various types of HRWRAs available, such as those based on polynaphthalene sulfonate (PNS), polymamine sulfonate (PMS), or lignosulfonate (LS) admixtures, as well as the most recently adopted polycarboxylate-based (PC) superplasticizers [18]. PC-based superplasticizers are widely adopted because of the way they disperse cement particles and retain slumps in concrete without increasing its setting time at a relatively low dosage [19]. PNS, PMS, and LS types of superplasticizers achieve the purpose of ensuring workability in cementitious materials through electro-steric means of dispersing cement particles, while PC superplasticizers, on the other hand, disperse cement particles by creating a steric barrier, which is essential to counter the dispersion forces (also known as Van Der Waals forces). These forces are responsible for the agglomeration of cement particles in SCCs, which subsequently leads to poor flow properties. O. Boukendakdji et al. [20] explained that PC-based superplasticizers’ dispersion mechanism is linked to the steric hindrance effect induced by the presence of long, neutral side graft chains.

PC-based superplasticizer molecules have a comb-like structure and are composed of a polyethylene main chain, polyethylene glycol side chains, and carboxylate functional groups [21,22,23]. Therefore, the chemical structure or molecular structure of PCs, among other factors such as chain order, is directly related to their potency on cementitious materials. Previous findings have shown that modifying the chemical structure of PC-based superplasticizers is easier to achieve in comparison to other forms of superplasticizers [24,25]. This ease of modification makes it possible to achieve the desired performance of SCCs. Furthermore, the ease of modification allows for the creation of various types of PC-based superplasticizers, examples of which are polycarboxylate-ester (PCE)-type and polycarboxylate-acid (PCA)-type superplasticizers. Studies have shown that polycarboxylate-based superplasticizers with different chemical structures perform differently in SCCs [24], so the difference in performance of the two types of polycarboxylates mentioned above is worth noting. Rixom et al. [17] discovered that polycarboxylate-ester (PCE)-type molecular compositions had more side chains, which improved slump retention, and fewer anionic binding sites to capture cement particles, while polycarboxylate-acid (PCA)-type molecule structures contain sufficient binding sites that make room for a sufficient dispersion of cement particles, which in turn enhanced the flow of SCCs with the addition of a small dosage. In support of this, Diawara and Ghafoori [15] noted that the higher the content of acid in a PCA-based superplasticizer, the easier it is for the admixture to adsorb cement particles, which eventually leads to a higher dispersibility. In contrast, the side chain concentration increases with an increasing ester ratio in a PCE-based superplasticizer, which lowers the carboxylic group content and reduces adsorption and dispersibility. In another study, Barfield and Ghafoori [26] concluded that polycarboxylate-ester-type superplasticizers needed a large dosage to achieve workability in their SCC mixtures when compared to the polycarboxylate-acid type. According to Aniewska-Piekarczyk [27], the properties vary based on the type of HRWRA used in the study. This author further explained that the SCC mixtures that require a lower dosage are those based on acrylates, modified phosphate, and HRWRAs based on polycarboxylates. However, the author did not use polycarboxylate-ester-type or polycarboxylate-acid-type superplasticizers in the study. HRWRAs’ effects on SCCs are influenced by their types and the conditions of their synthesis. Because of this, even if the fundamental design and action of HRWRAs are identical, the impact of their chemical structure on performance can vary depending on the manufacturer or source.

Łaźniewska-Piekarczyk [27] carried out an investigation on the effects of various sources of superplasticizer on the void content and flowability of SCCs. It was revealed that, despite their apparent chemical similarity, admixtures from different sources cannot be used alternately. In another study, Łaźniewska-Piekarczyk [28,29] investigated the effect of different sources of HRWRAs on the air content in cement paste and the fresh properties of SCCs. Due to its “air-entraining” effect on the SCC mixture and the requirement of the superplasticizer compatibility test as a complement to validate the effect of “air-entrainment”, the test result highlighted the significance of the source of the superplasticizer. The author also concluded that, regardless of the yield stress and viscosity of the SCC, the type of superplasticizer considerably affects the amount of air in a self-compacting mix. Barfield and Ghafoori [26] also observed significant differences between the admixture sources required to obtain the optimum dosage rate necessary for the required fresh properties and the air-void characteristics of freshly mixed SCCs.

## 2. Research Objective

In the study presented herein, the effects of different polycarboxylate-based superplasticizers obtained from four distinct manufacturing sources on the fresh properties of SCC were investigated, and the study compared the optimum (minimum) dosage requirements of the four different sources of polycarboxylate-based high-range water reducers and viscosity-modifying admixtures in attaining slump flows of 508 mm, 635 mm, and 711 mm.

## 3. Materials and Methods

### 3.1. Raw Materials

ASTM C150 [30] Type V Portland cement, ASTM C618 [31] Class F fly ash, and locally produced coarse and fine aggregates were used as the matrix constituents. The Type V Portland cement had a Blaine fineness of 423 m^2^/kg and the following percentages of chemical constituents: SiO_2_ = 20.1%; Al_2_O_3_ = 4.0%; Fe_2_O_3_ = 3.6%; CaO = 63.5%; MgO = 2.8%; SO_3_ = 2.9%; C_3_A = 4%; C_3_S = 58%; C_2_S = 14%; Na_2_O equivalent = 0.57%. Its loss on ignition was 2.3% and insoluble residue was 0.44%. The fly ash had the following chemical composition: SiO_2_ = 58.2%; Al_2_O_3_ = 17.4%; Fe_2_O_3_ = 4.8%; CaO = 7.9%; SO_3_ = 0.6%. Its moisture content was 0.0% and loss on ignition was 4.2%. The fine aggregate met the ASTM C33 [32] requirements. Its bulk and saturated surface dry specific gravity, absorption, and fineness modulus were 2.75, 2.78, 0.8%, and 3.0, respectively. The coarse aggregate had a nominal maximum size equal to 12.50 mm and complied with ASTM C33 [32] size number 7. Its bulk and saturated surface dry specific gravity, absorption, and dry rodded unit weight were 2.77, 0.6%, 1634 kg/m^3^, respectively. Other concrete constituents were tap water, polycarboxylate-based HRWRA, and VMA complying with the ASTM C494 Type F requirements [33].

### 3.2. Mixing Procedure and Testing

An electric counter-current pan mixer with a capacity of 0.028 m^3^ was used to blend the concrete components at a rate of 14.5 rpm. Batch volumes of 0.017 to 0.023 m^3^ were used for all mixtures. The mixing sequence consisted of blending the coarse aggregate with 1/3 of the mixing water for two minutes, followed by the fine aggregate with 1/3 of the mixing water for another two minutes, and the cementitious materials with the remaining 1/3 of the mixing water for three minutes. Finally, the HRWRA and VMA were added and the blending of the matrix continued for an additional three minutes, followed by a two-minute rest and resumption of mixing for two additional minutes.

The freshly mixed self-consolidating concretes were used to determine the unconfined workability, flow rate/plastic viscosity, passing ability, filling ability, and static and dynamic stabilities using slump flow, T_50_, J-ring, U-box and L-box, V-funnel, and visual stability index (VSI) and column segregation tests, respectively [34,35,36,37]. The tests on the fresh concretes were conducted immediately after mixing to avoid any variations over time. Each mixture was repeated at least three times, and the reported test results reflect the average value of a minimum of three tests.

## 4. Results and Discussion

### 4.1. Optimum Admixture Dosage

**Figure 1:**
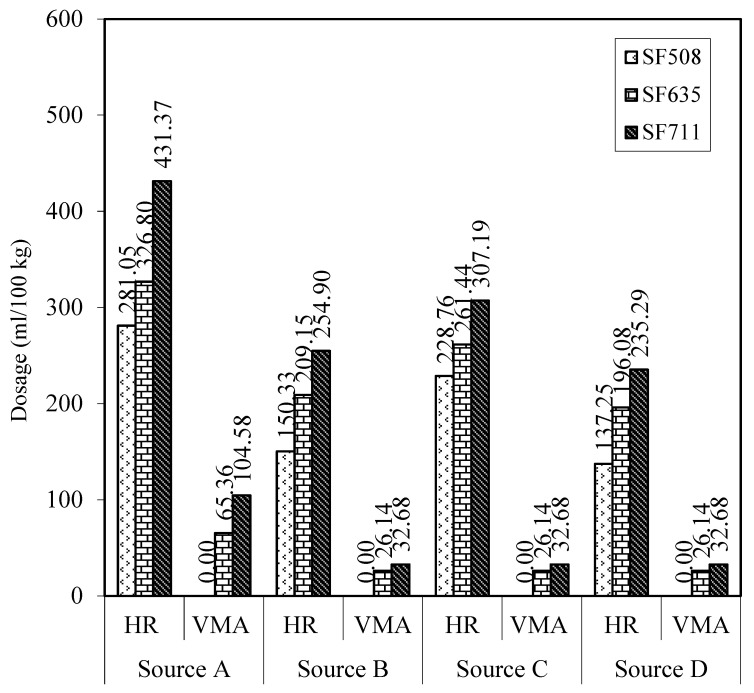
Optimum admixture dosages.

The optimum admixture dosage was defined as the minimum amount of admixture required to achieve the target unconfined workability and dynamic stability. The optimized dosage requirements of the HRWRA and VMA from the four selected admixture sources are presented in Table 1. The comparisons of the test results are shown in Figure 1. The discussion of the optimum admixture dosage, as influenced by the admixture source and slump flow, is presented in the following sections.

#### 4.1.1. Influence of Admixture Source on Optimum Admixture Dosage

Figure 1 shows the influence of the four selected admixture sources on the optimum admixture dosage to attain the target slump flows of 508 mm, 635 mm, and 711 mm, and a VSI of 0 or 1. The test results indicate that there were differences in the dosage requirement for the HRWRA and VMA in meeting the above-mentioned fresh properties. The required dosage amount of the HRWRA was highest for source A, followed by sources C, B, and D (in descending order). The optimum dosages of sources B, C, and D superplasticizers in making the 508 mm SCC slump flow were 47, 19, and 51% lower, respectively, when compared to that of source A. The corresponding reductions in the optimum HRWRA dosages were 36, 20, and 40% and 41, 29, and 45% for 635 and 711 mm slump flows, respectively. When compared to the admixture source A, the reductions in the VMA dosage for sources B, C, and D were fairly uniform at about 60 and 69% for the SCCs with 635 and 711 mm slump flows, respectively. All 508 mm slump flow self-consolidating concretes displayed acceptable dynamic stability and plastic viscosity without the use of the viscosity-modifying admixture.

The information concerning the exact chemical structure and molecular weight of the HRWRA and VMA used in this investigation could not be obtained from the manufacturers. The variations in the optimum admixture dosages amongst the four HRWRA sources were studied through evaluations of (a) the absorbance of the HRWRA molecules in cement–water–HRWRA solutions obtained using ultraviolet–visible (UV–Vis) spectroscopy tests and (b) the mechanical action of the viscosity-modifying admixtures (VMA) and the VMA-to-HRWRA ratio.

##### Evaluation of Adsorption of HRWRAs Using UV–Vis Spectroscopy

This section is intended to explain and confirm the test results related to the trend of the optimum dosage requirements of the selected polycarboxylate-based HRWRAs (PC-HRWRAs). In general, all four admixture sources have the same mechanism of action, namely adsorption, electrostatic repulsion, and steric repulsion. The PC-HRWRA carboxyl group (COO^−^) must be adsorbed first to the cement calcium ions (Ca^2+^) before they are able to play a dispersing role. The UV–Vis test was used to evaluate the concentration of free admixture in the cement–water–HRWRA solution before a correlation with admixture adsorption could be made. The relationship between the increase in concentration of free admixture and the increase in adsorption amount was established through the effect of the slump flow on the admixture dosage. As can be seen in Figure 1, a higher slump flow required a higher dosage of the admixture.

UV–Vis spectroscopy absorption is not a specific test for any given compound. The nature of the solvent, the pH of the solution, temperature, high electrolyte concentrations, and the presence of interfering substances can influence the absorption spectra of compounds, as can variations in the effective bandwidth of the spectrophotometer [38]. However, the wavelengths of absorption peaks can be correlated with the types of bonds in each molecule and are valuable in determining the functional groups within a molecule [38]. The experiment used a test sample in the UV–Vis beam to determine the absorbance or transmittance at different wavelengths. Alternatively, samples were prepared in known concentrations and their absorbance was determined using the UV–Vis spectrophotometer. The results were then graphed to make a calibration curve from which the unknown concentration can be determined based on its absorbance.

**Figure 2:**
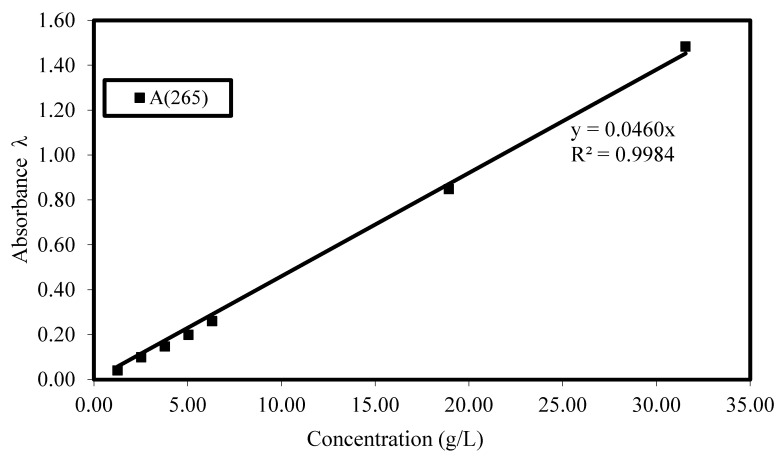
Calibration curve of source C HRWRA at wavelength of 265 nm.

In the present investigation, a uniform cement content of 390 kg/m^3^ and a constant water-to-cementitious materials ratio of 0.4 were used for all trial matrices. The dosage of the HRWRA was kept constant at 255 mL/100 kg for all four admixture sources, and distilled water was used throughout the study to avoid any contamination which could impair the test results. The test procedure was as follows:

First, the calibration curves for interpolation were generated. For that purpose, the selected polycarboxylate-based HRWRAs were manually diluted in distilled water at different concentrations. After 10 min, the solutions were analyzed by the UV–Vis spectroscopy, and calibration curves of known HRWRA concentrations as a function of the recorded absorbance were plotted. Figure 2 presents a typical calibration curve. The test results indicated a very strong relationship between the concentration of the HRWRA in water and the recorded absorbance, as indicated by the coefficient of multiple determinations (R^2^).

**Figure 3:**
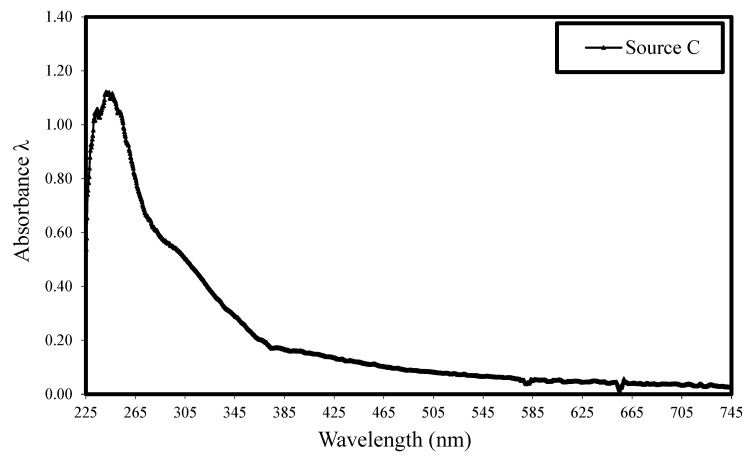
Typical ultraviolet–visible absorbance spectrum of source C HRWRA.

**Figure 4:**
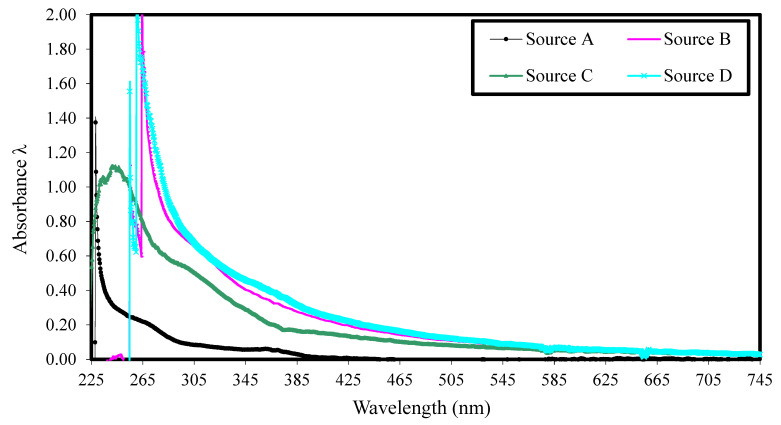
Comparison of ultraviolet–visible absorbance spectra of HRWRAs from sources A, B, C, and D.

Second, UV–Vis absorption curves for the cement–water–HRWRA solution were made. The cement and water were first mixed in a pan mixer at 14.5 rpm for 5 min before the pre-measured HRWRA was added and afterward, the mixing continued for an additional 5 min. The blended paste was placed in sterilized tubes and centrifuged by ultracentrifugation for 5 min at 3500 rpm to suspend the fine particles in solution. The liquid at the top of the sample was collected with a pipette and transferred into a syringe mounted on a 0.20 μm filter. The filtered liquid was then tested by UV–Vis spectroscopy. Figure 3 shows typical ultraviolet absorption spectra and Figure 4 displays the UV–Vis absorption spectra of the four selected admixture sources.

The test results shown in Figure 4 indicated that the recorded absorbance peaks varied from one admixture to another as they occurred at different wavelengths (from 230 to 265 nm), indicating the differences in chemical type. The recorded results were also used to determine the actual concentration of free admixture in the liquid phase of the cement–water–HRWRA solution. The calculated concentrations of the selected admixture sources are summarized in Table 2. The solution concentration of the free admixture was highest for source D, followed by sources B, C, and A (in descending order). In general, the differences between the HRWRAs from sources B, C, and D were relatively small, indicating that they might have similar chemical structures. On the other hand, the HRWRA from source A produced results that were different than those from sources B, C, and D.

The review of the related literature provided by the manufacturers indicated that the four selected superplasticizers are either a polycarboxylate-ester or polycarboxylate-acid type. The behaviors of the superplasticizers from sources B, C, and D were similar to that of a polycarboxylate-acid type, where the acid portion is predominant when compared to the ester part. The superplasticizer from source A had the highest ester-to-acid ratio and was a polycarboxylate-ester type. The higher the acid ratio is, the higher the carboxylic group content is, and the higher the adsorption ability is. When the ester ratio is predominant, the side chain content increases and the carboxylic group content decreases, leading to a decrease in adsorption and dispersibility. This finding also confirms the findings for the optimum dosage requirements of the four selected admixture sources reported earlier.

##### Mechanism of Action of VMA

The difference in the optimum dosage of the viscosity-modifying admixture from the four sources can be attributed to the mechanism by which these admixtures function. Based on the reported results obtained during this study, the viscosity-modifying admixture from source A required a higher optimum VMA dosage than those from sources B, C, and D. The source A viscosity-modifying admixture functioned by thickening the concrete, making it very cohesive without significantly affecting the fluidity of the fresh matrix. The viscosity-modifying admixture from sources B, C, and D functioned by binding the water within the concrete mixture, resulting in an increase in viscosity while reducing or eliminating concrete bleeding. The present investigation revealed that a large amount of the source A VMA was always needed to modify the viscosity of the SCC, while a small amount of the VMAs from sources B, C, and D generated a noticeable improvement in the fresh performance of the selected self-consolidating concretes.

##### VMA-to-HRWRA Ratio

The minimum dosage required to achieve the target fresh properties was obtained by trial-and-error combinations of the HRWRA and VMA. The analysis of the test results obtained during this study indicated a trend for the VMA-to-HRWRA ratio, as can be seen in Table 3. The similarity in the VMA-to-HRWRA ratios of the admixtures from sources B, C, and D further affirmed that these admixtures have similar chemical compositions. The higher ratio seen in the admixture from source A was due to its thickening mode of action, which led to a higher amount of VMA required to make highly stable or stable matrices.

#### 4.1.2. Influence of Slump Flow on Optimum Admixture Dosage

As shown in Table 1 and Figure 1, the required admixture dosages increased with an increase in slump flow, regardless of the admixture source and the selected SCC group. For the selected self-consolidating concretes, as the slump flow increased from 508 to 635 and 711 mm, the optimum amount of the HRWRA increased by 16 and 32%, 39 and 22%, 14 and 18%, and 43 and 20% for the admixtures from sources A, B, C, and D, respectively. The increases in the VMA dosage remained at 60% for the admixture from source A, and 25% for the admixtures from sources B, C, and D when the slump flow changed from 635 to 711 mm. No VMA was needed for self-consolidating concretes with a 508 mm slump flow.

The increase in the optimum dosage requirement for the HRWRA and VMA to obtain a higher slump flow can be explained through the demand in the rheological performance of the concrete. During the deflocculation system, the bond between the finer cement particles was gradually broken by the mixing water until a uniform matrix (normal slump concrete) was generated. From that moment, a superplasticizer was needed to produce a flowable matrix. The need for a higher slump flow required an increase in the amount of the HRWRA. In the presence of a higher amount of HRWRA, the force needed to disperse the ingredients of the fresh matrix, i.e., the yield stress, was gradually reduced as the fresh concrete was allowed to spread further (HRWRAs reduce the yield stress by dispersing cement particles and increasing the electrostatic repulsion, leading to a more flowable concrete. VMAs help maintain mix stability, ensuring that the concrete does not segregate despite its improved flowability). In fact, when the amount of superplasticizer was increased, the adsorbed amount of polymer molecules on the cement particles increased along with the induced zeta potential (the potential difference between the dispersion medium and the stationary layer of fluid attached to the dispersed particle), leading to higher electrostatic repulsion forces. Additionally, the intensity of the steric repulsive forces (which are short-range repulsive forces caused by the overlapping of the adsorbed polymer) was also increased when a higher HRWRA dosage was used.

The increase in the slump flow value or HRWRA dosage was usually accompanied by a decrease in plastic viscosity, and a viscosity-modifying admixture was needed to overcome that problem. The addition of a VMA restored the plastic viscosity that was deteriorated by the increase in the HRWRA dosage.

### 4.2. Fresh Characteristics

The results for the fresh characteristics of the selected self-consolidating concretes are shown in Table 4. The discussion of the fresh performance of the selected self-consolidating concretes as related to their flowability, viscosity, stability, passing ability, and filling ability is presented below.

#### 4.2.1. Flowability/Viscosity

Slump flow values were used to describe the flowability of the fresh concrete in an unconfined condition, and the slump flow (SF) test is the preferred test method for flowability. The flowability of a given fresh SCC is related to its viscosity. The flow times of T_20_, T_40_, and T_50_ and V-funnel flow time can be used to measure both the flowability and the viscosity.

##### Influence of Admixture Source on Flowability/Viscosity

The test results indicate that for slump flows of 508, 635, and 711 mm, the selected self-consolidating concretes made with source B and C admixtures displayed similar T_50_ times which were on average 15, 21, and 16% higher, respectively, than those of the concretes prepared with the admixtures from sources A and D. For the V-funnel test results, the admixtures from sources B and C displayed on average 6, 5, and 3% reductions in t_v_ when compared to the admixtures from sources A and D for the SCCs prepared with slump flows of 508, 635, and 711 mm, respectively.

In summary, it can be concluded that the T_50_ time and V-funnel flow time varied amongst the selected self-consolidating concretes and were all within the acceptable values recommended by the ASTM committee C09.47 [34]. The admixtures from sources B and C displayed similar flowability which was lower than that from sources A and D, or, by inference, the admixtures from sources B and C showed a higher viscosity when compared to those from sources A and D.

##### Influence of Slump Flow on Flowability/Viscosity

The increase in the flowability of the selected self-consolidating concretes led to reductions in the T_50_ and V-funnel flow times. For the designed SCCs, when the slump flow increased from 508 to 635 and 711 mm, the T_50_ and V-funnel flow times decreased on average by 16 and 10%, and 19 and 8%, respectively. This, by no means, is a statistically rigorous comparison, but it gives a good idea of the trend in flowability/viscosity changes with an increase in slump flow values. The decrease in viscosity (or increase in flowability) induced by an increase in slump flow can be attributed to increases in the adsorption of the admixture, leading to an increase in dispersion of cement flocs and the breakdown of the bond between the cement particles due to increases in the amount of superplasticizer. The incorporation of a VMA helped to partially restore the loss in viscosity by elevating the T_50_ and V-funnel flow times to acceptable values.

#### 4.2.2. Stability

Both the dynamic and static stabilities of the trial self-consolidating concretes were evaluated. The results are presented below.

##### Dynamic Segregation Resistance

Dynamic segregation resistance was evaluated by visual examination of the fresh concrete and reported as a VSI score. A visual assessment for any indication of mortar/paste separation at the circumference of the flow and any aggregate separation in the central area gives an indication of the dynamic segregation resistance.

##### Influence of Admixture Source on Dynamic Segregation Resistance

All selected self-consolidating concretes were designed to attain a visual stability index of 0 (highly stable concrete) or 1 (stable concrete) by balanced the proportions of the HRWRA and VMA once a sufficient cementitious material content and an appropriate coarse-to-fine aggregate ratio were obtained. As reported in Table 4, irrespective of the admixture source, the target VSI of 0 or 1 was obtained for all trials matrices. No evidence of segregation or bleeding in slump flow was observed in any of the selected self-consolidating concretes, indicating that stable matrices were attained with all four admixture sources.

##### Influence of Slump Flow on Dynamic Segregation Resistance

Highly stable mixtures (VSI = 0) were achieved for the selected self-consolidating concretes with 508 mm and 635 mm slump flows. When the slump flow was increased from 635 to 711 mm, the attainment of a highly stable matrix was not possible without the utilization of an excessive and impractical amount of the admixtures. Consequently, to maintain a practical design while searching for the optimum dosage and proportioning of the admixtures, a ranking of the stable dynamic segregation resistance (VSI = 1) was adopted for the 711 mm slump flow self-consolidating concretes. The HRWRA and VMA were used in the selected concretes to decrease their yield stress and increase their plastic viscosity, respectively. The reduction in dynamic stability for the 711 mm slump flow self-consolidating concretes was primarily due to the increase in the amount of HRWRA, leading to a gain in dispersibility and a reduction in the homogeneity of the matrix. Irrespective of the admixture source, the selected self-consolidating concretes made with 635 mm and 711 mm slump flows required the use of a VMA to obtain an acceptable visual stability index.

##### Static Segregation Resistance

In this investigation, the static segregation resistance of self-consolidating concrete was determined using column segregation tests. The top-to-bottom retained #4 sieve coarse aggregate mass (weight) ratio was measured to find the segregation resistance of the SCC [37]. This section discusses the static stability of the designed self-consolidating concretes as related to the admixture source and slump flow.

##### Influence of Admixture Source on Static Segregation Resistance

The segregation indices (SIs) of the trial matrices, as reported in Table 4, were lower than the maximum recommended value of 15%. For the selected SCCs, the admixture from sources A and D exhibited similar segregation indices which were higher than those of the admixtures from sources B and C. This is indicative of a higher static segregation resistance of the SCCs made with the admixtures from sources B and C compared to those made with the admixtures from sources A and D. On average, the matrices incorporating the admixtures from sources A and D experienced reductions in static stability of 20 and 26%, respectively, when compared to those obtained when the admixtures from sources B and C were used. The increase in static segregation resistance due to the admixtures from sources B and C may be attributed to their higher viscosity (by inference), as can be seen from the results of the T_20_, T_40_, T_50_, and V-funnel times reported in Table 4.

##### Influence of Slump Flow on Static Segregation Resistance

The segregation indices of the selected self-consolidating concretes increased as the slump flow increased, irrespective of the admixture source. When the slump flows increased from 508 to 635 and 711 mm, the static stability decreased on average by 23 and 27%. This was mainly due to the reduction in the viscosity (by inference) of the higher slump flow concrete.

The static stability mechanism of action can be explained through aggregate sedimentation, which is related to the viscosity and the density of the mixture, the size and the density of the aggregate, and the flow velocity of the mixture.

#### 4.2.3. Passing Ability

The passing ability or the capacity of the fresh matrix to flow through confined spaces and narrow openings without blocking was measured using the J-ring, L-box, and U-box tests. For an acceptable SCC, a J-ring value between 0 and 51 mm (0 and 2 inches), an L-box flow height ratio H_2_/H_1_ of 0.8 to 1, and a U-box filling height H_1_-H_2_ lower than 305 mm (12 inches) are recommended. The current section is intended to discuss the influence of the four selected admixture sources and the three slump flow values on the passing ability of the designed self-consolidating concretes.

##### Influence of Admixture Source on Passing Ability

As shown in Table 4, the measured J-ring values of the admixtures from the four sources were between 25 and 50 mm, indicating a moderate passing ability (passing ability rate of 1) or minimal to noticeable blocking of the selected self-consolidating concretes.

The test results related to the L-box and U-box tests are also presented in Table 4. The flow height ratios H_2_/H_1_ of the 508 mm slump flow self-consolidating concretes were less than the minimum recommended value of 0.8, indicating their extreme blocking ability. However, regardless of the admixture source, for concretes made with a slump flow of 635 or 711 mm, the flow height ratios remained near the bottom third of the recommended limits, indicating their moderate passing ability.

The results pertaining to the U-box test were also indicative of a moderate passing ability for the selected SCC mixtures. The U-box filling height H_1_-H_2_ values of the selected SCCs were near the upper limit of the allowable 305 mm value.

Overall, with proper proportioning, self-consolidating concretes with an acceptable passing ability can be achieved with any of the admixtures from the four selected sources.

##### Influence of Slump Flow on Passing Ability

Irrespective of the admixture source, the passing ability of the selected matrices improved with an increase in slump flow. When the slump flow increased from 508 to 635 and 711 mm, the J-ring passing ability improved by an average of 14 and 14%. Similar gains in passing ability were observed when the assessment included the L-box or U-box tests. The corresponding improvements in the L-box passing ability were 28 and 5%, and 5 and 8%, respectively, when the U-box test was used. This behavior can be attributed to a decrease in the yield point and an increase in the viscosity of the higher slump flow self-consolidating concretes (as shown by T_50_), allowing ease of movement around blocking rebars.

#### 4.2.4. Filling Ability

V-funnel and U-box tests were also utilized to assess the filling ability of the selected concretes. As reported above, the test results for the admixtures from all four sources (the V-funnel times and U-box filling heights) were indicative of their moderate filling ability for the selected SCCs.

### 4.3. Predictive Statistical Equations and Ranking of the SCC Admixture Dosage

The purpose of this statistical analysis is to show the existence of a relationship among the selected variables and to demonstrate the significance of selected dependent variables. The predictive statistical analysis given below is valid only for the present study.

As it can be seen in Table 1, the selected trial matrices have different proportions of paste, in terms of the volume ratio (P), mortar volume ratio (M), and coarse aggregate absolute volume (C_Aggr_). The matrix factor *β* = *P*·*M*·C_Aggr_ was used to characterize each trial matrix. The equations to predict the optimum admixture dosage requirement were determined using a statistical program [39]. Analyses were conducted at a 95% confidence level. The predictive equations were tested for accuracy using R² (the coefficient of multiple determination) and S (average standard deviation). Correlations between the data predicted from the regression equations and the actual test results were evaluated using F and T tests. Due to the difference in their mechanism of action, the admixture from source A and the admixture from sources *B*, *C*, and *D* were analyzed separately. The optimum HRWRA and the VMA dosages were related to the target slump flow and the matrix factor (*β*) through the following equations:

Admixture from source A

(1) $$HR_{A}=188,148.26+0.7386SF-\frac{28,390.11}{\beta }+\frac{1070.24}{\beta^{2}}$$

(2) $$VMA_{A}=883,556.62+0.9014SF-\frac{134,854.26}{\beta }+\frac{5141.37}{\beta^{2}}$$

Admixtures from sources *B*, *C*, and *D*

(3) $$HR_{B,C,D}=75,080,212.01-\frac{45,592.27}{SF}-\frac{17,184,249.17}{\beta }+\frac{1,310,935.51}{\beta^{2}}-\frac{33,333.10}{\beta^{3}}$$

(4) $$\begin{array}{c} VMA_{B}=88,468.88+\frac{1,979,137.54}{SF}-2,369,377.96\beta -\frac{122,606,642.61}{SF^{2}}\\ +15,826,854.10\beta^{2}-21,814,887.75\frac{\beta }{SF} \end{array}$$

where

*HR_A_*, *HR_B_*_,*C*,*D*_ = optimum dosage of high-range water-reducing admixture (mL/100 kg);

*VMA_A_*, *VMA_B_*_,*C*,*D*_ = optimum dosage of viscosity-modifying admixture (mL/100 kg);

*SF* = expected slump flow (mm), with 508 mm ≤ *SF* ≤ 711 mm (tolerance ± 25 mm);

*β* = *P*·*M*·C_Aggr_ (%)

where

*P* = paste volume ratio;

*M* = mortar volume ratio;

C_Aggr_ = coarse aggregate absolute volume.

N.B.:

− The paste and mortar used in β do not include the admixtures.
− No VMA is needed for a slump flow SF ≤ 508 mm.

The regression variables R², S, Prob(t), and Prob(F) are given in Table 5. The calculated values are indicative of a strong relationship between the dependent variable (HRWRA or VMA) and the independent variables (slump flow value, paste volume ratio, mortar volume ratio, and coarse aggregate absolute volume). The predictive equations yielded percentage errors ranging, for most part, from 0 to 10%, confirming a good relationship between the actual and the predicted optimum admixture dosages.

The ranking of the four admixture sources in terms of their influence on the fresh performance of the trial self-consolidating concrete is presented in Table 6.

### 4.4. Compressive Strength

The ASTM C 39, “Standard Test Method for Compressive Strength of Cylindrical Concrete Specimens”, was used to evaluate the compressive strength of the designed self-consolidating concretes. The compressive strength test results of the selected trial mixtures at different curing ages are shown in Table 7. Each of these values is the average of four tested cylinders.

#### 4.4.1. Influence of Admixture Source on Compressive Strength

**Figure 5:**
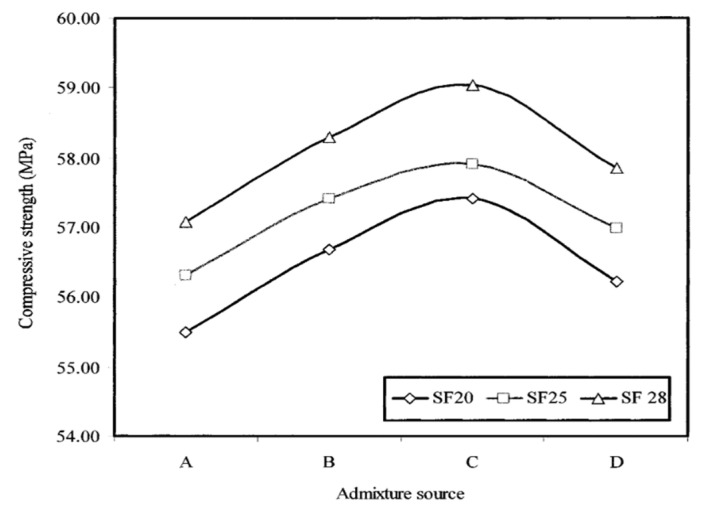
Influence of admixture source on the 28-day compressive strength of self-consolidating concretes.

**Figure 6:**
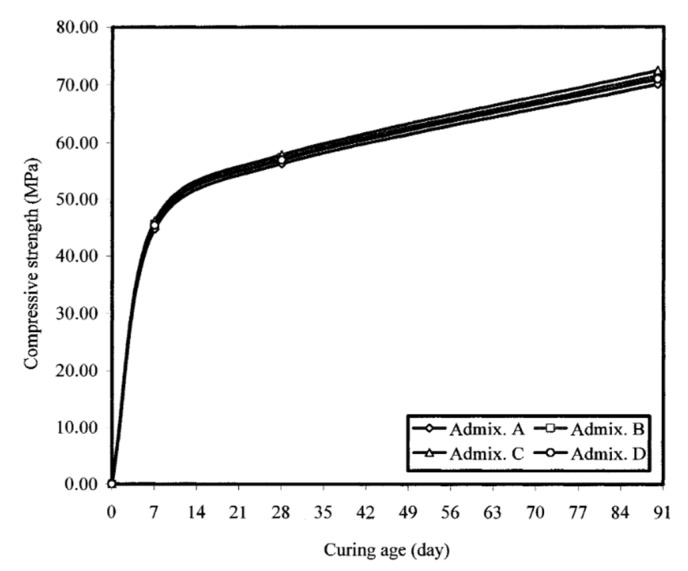
Influence of admixture source and curing age on the compressive strength of 635 mm (25 inches) slump flow self-consolidating concretes.

**Figure 7:**
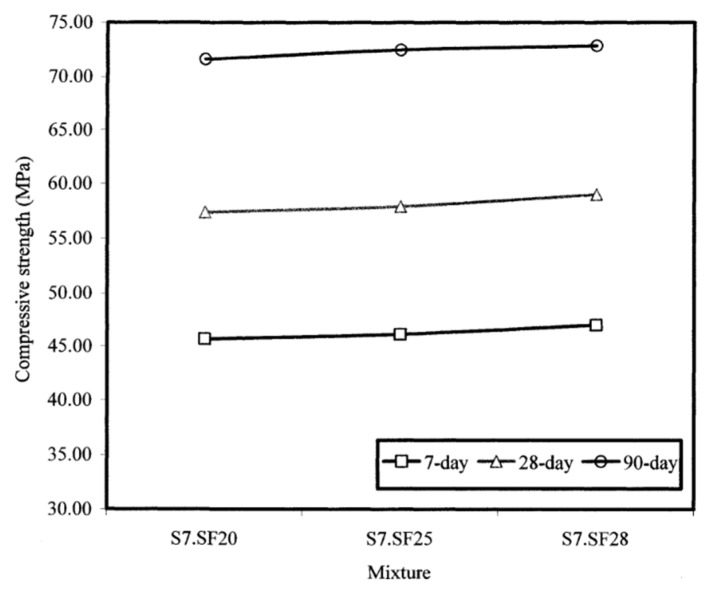
Influence of slump flow value on the compressive strength of self-consolidating concretes made with admixture from source C.

Typical representations of compressive strength as a function of admixture source and curing age are displayed in Figure 5, Figure 6 and Figure 7, respectively. In comparison to the admixture from source C, the studied self-consolidating concretes incorporating admixtures from sources A, B, and D showed reductions in compressive strength of 3, 1, and 2%, respectively, regardless of the slump flow and curing age. These relatively small variations indicate that the four selected polycarboxylate-based HRWRAs and their corresponding VMAs had the type of chemical composition that did not interfere with the hydration reaction and did not alter the compressive strength development of the concrete. The increase in strength at 28 and 90 days was attributed to the availability of more calcium silicate hydrate (C-S-H) binder due to the pozzolanic reaction of the fly ash with lime, and the continued hydration of the cement paste.

#### 4.4.2. Influence of Slump Flow on Compressive Strength

Figure 7 is a typical representation of the compressive strength as a function of the slump flow. On the whole, when the slump flow increased from 508 to 635 to 711 mm (20 to 25 to 28 inches), irrespective of the admixture source and curing age, all three SCC groups displayed similar compressive strength improvements of less than 3% variation. This marginal difference in compressive strength indicated the insignificant influence of the increased fluidity of the self-consolidating concrete due to increases in the slump flow through higher dosages of admixtures.

#### 4.4.3. Effect of Admixture Source on the Strength of the Concrete

Compared to the admixture from source C, the studied self-consolidating concretes incorporating admixtures from sources A, B, and D showed reductions in compressive strength of 3%, 1%, and 2%, respectively, regardless of the slump flow and curing age. These relatively small variations indicate that the four selected polycarboxylate-based high-range water-reducing admixtures (HRWRAs) and their corresponding viscosity-modifying admixtures (VMAs) had chemical compositions that did not interfere with the hydration reaction or alter the compressive strength development of the concrete. The increase in strength at 28 and 90 days was attributed to the availability of more calcium silicate hydrate (C-S-H) binder, resulting from the pozzolanic reaction of the fly ash with lime and the continued hydration of the cement paste.

## 5. Summary of the Results

HRWRA Dosage:

- Source A required the highest dosage amount of the HRWRA, followed by sources C, B, and D (in descending order).
- The optimum dosages of sources B, C, and D superplasticizers were 47%, 19%, and 51% lower, respectively, compared to source A for a 508 mm slump flow.
- The reductions in the HRWRA dosage for sources B, C, and D were 36%, 20%, and 40% for a 508 mm slump flow.
- The increases in the HRWRA dosage ranged from 14% to 43% as the slump flow increased from 508 to 711 mm for all admixture sources.

VMA Dosage:

- The reductions in the VMA dosage for sources B, C, and D were fairly uniform at about 60% and 69% for SCCs with 635 mm and 711 mm slump flows, respectively.
- The increase in the VMA dosage remained at 60% for source A and 25% for sources B, C, and D when the slump flow changed from 635 to 711 mm.
- No VMA was needed for SCCs with a 508 mm slump flow.

Visual Stability Index (VSI):

- The study aimed to achieve a VSI of 0 or 1, indicating good visual stability.
- The proper proportioning of admixtures led to self-consolidating concretes with an acceptable flowability, plastic viscosity, dynamic and static stabilities, passing ability, and filling ability for all four selected admixture sources

## 6. Conclusions

Based on the test results of this study, the following conclusions can be drawn:

(a) Irrespective of the self-consolidating concrete group, the optimum dosage requirements to obtain a uniform slump flow and visual stability index varied amongst the four selected admixture sources. An increase in the slump flow generated a higher dosage demand for all admixture sources.
(b) The differences among the admixture sources can be explained through the adsorption amount of the HRWRA molecules on cement grains, the chemical type/bonds of the HRWRA, and the calculated VMA-to-HRWRA ratio. The behaviors of the superplasticizers from sources B, C, and D were similar to that of a polycarboxylate-acid (PCA) type, whereas the acid portion was predominant when compared to the ester part. On the other hand, source A was a polycarboxylate-ester type and at the same dosage, it was unable to disperse cement grains at the same as polycarboxylate-acid types.
(c) With proper proportioning, self-consolidating concrete with an acceptable flowability, plastic viscosity, dynamic and static stabilities, passing ability, and filling ability can be achieved with any of the four selected admixture sources. However, the performance of the selected admixtures in attaining uniform fresh properties varied among the admixture sources.
(d) The 508 mm slump flow SCCs exhibited a very low plastic viscosity (based on inference), very high dynamic stability, moderate filling ability, low passing ability, and high static stability. As a result, a 508 mm slump flow was found to be unsuitable for congested reinforced structures. All 635 mm and 711 mm slump flow self-consolidating concretes displayed a high flowability, low plastic viscosity (by inference), high dynamic stability, moderate static stability, moderate passing ability, and moderate to high filling ability, indicating their suitability for most civil engineering applications. The formwork for the 711 mm slump flow SCCs may be subjected to a higher-than-expected pressure due to the flowability that remained near the lower bond of the acceptable limit.
(e) The predictive equation to correlate the dependent variable (HRWRA or VMA) with independent variables (past content, aggregate size, and target slump flow) showed significant statistical relationships.
(f) This study showed that self-consolidating concretes with admixtures from sources A, B, and D showed only slight reductions in compressive strength (1–3%) compared to source C, suggesting that the admixtures do not significantly interfere with hydration or strength development. The strength increases at 28 and 90 days were due to the pozzolanic reaction of the fly ash and continued cement hydration.

## Figures and Tables

**Table 1:** Proportion of components in self-consolidating concretes.

Mix No.	Portland Cement (kg/m^3^)	Fly Ash (kg/m^3^)	w/cm ^1^	Fine Aggre. (kg/m^3^)	Coarse Aggre. (kg/m^3^)	Admixture Dosage (mL/100 kg)		Paste Fraction	Mortar Fraction	Volume of Coarse Aggre.
						HRWRA ^2^	VMA ^3^	(%)	(%)	(%)
A. SF508	390	78	0.40	849	922	281.05	0.00	34.60	65.62	33.04
B. SF508	390	78	0.40	849	922	150.33	0.00	34.55	65.63	33.03
C. SF508	390	78	0.40	849	922	228.76	0.00	34.58	65.63	33.03
D. SF508	390	78	0.40	848	922	137.25	0.00	34.54	65.63	33.03
A. SF635	390	78	0.40	849	922	326.80	65.36	34.63	65.62	33.05
B. SF635	390	78	0.40	849	922	209.15	26.14	34.58	65.62	33.04
C. SF635	390	78	0.40	849	922	261.44	26.14	34.60	65.62	33.04
D. SF635	390	78	0.40	849	922	196.08	26.14	34.57	65.63	33.04
A. SF711	390	78	0.40	849	923	431.37	104.58	34.69	65.61	33.05
B. SF711	390	78	0.40	849	922	254.90	32.68	34.60	65.62	33.04
C. SF711	390	78	0.40	849	922	307.19	32.68	34.62	65.62	33.04
D. SF711	390	78	0.40	849	922	235.29	32.68	34.59	65.62	33.04

^1^ water-to-cementitious materials ratio, ^2^ high range water reducing admixture, ^3^ viscosity-modifying admixture; 1 kg/m^3^ = 1.6856 lb./yd^3^, 1 mL/100 kg = 0.0153 oz/cwt; A, B, C, and D represent the selected four admixture sources; SF508, SF635, and SF711 stand for slump flows of 508, 635, and 711 mm, respectively.

**Table 2:** Cement–water solution’s concentration of free HRWRA.

Designation	* Absorbance (l, nm)			Increase in HRWRA Concentration (g/L)
	A (265)	A (700)	A (265 Corr)	
Source A	1.039	0.013	1.026	15.43
Source B	2.558	0.051	2.507	33.96
Source C	1.624	0.054	1.570	16.61
Source D	2.495	0.052	2.443	194.90

* Absorbance at wavelength l, in nanometers.

**Table 3:** VMA-to-HRWRA dosage ratios.

Admixture Source	Group I SCC		Group III SCC	
	Slump Flow 635 mm	Slump Flow 711 mm	Slump Flow 635 mm	Slump Flow 711 mm
A	0.59	0.78	0.20	0.24
B	0.19	0.20	0.13	0.13
C	0.17	0.19	0.10	0.11
D	0.17	0.16	0.13	0.14

1 mm = 0.03937 inch.

**Table 4:** Fresh properties of self-consolidating concretes.

Mix No.	Slump Flow (mm)	T_50_ (s)	VSI	J Ring Value (mm)	SI (%)	L Box H_2_/H_1_			U-Box H_1_-H_2_ (mm)	V-Funnel (s)
						H_2_/H_1_	T_20_ (s)	T_40_ (s)		
S7.A.SF20	527.05	2.69	0	43.94	6.37	0.65	0.55	1.62	225.55	4.82
S7.B.SF20	524.00	3.19	0	42.67	4.83	0.63	0.71	2.45	244.60	5.12
S7.C.SF20	524.00	3.15	0	45.72	5.07	0.65	0.67	1.82	247.65	5.17
S7.D.SF20	511.30	2.82	0	44.45	7.15	0.70	0.63	1.79	242.32	4.90
S7.A.SF25	651.00	2.48	0	36.83	8.12	0.84	0.52	1.61	215.90	4.35
S7.B.SF25	651.00	2.79	0	38.86	5.72	0.83	0.70	1.97	231.90	4.55
S7.C.SF25	651.00	2.69	0	37.59	5.64	0.83	0.59	1.74	231.65	4.65
S7.D.SF25	649.73	2.04	0	38.10	9.57	0.86	0.58	1.70	234.95	4.40
S7.A.SF28	727.20	1.85	1	31.75	9.11	0.88	0.48	1.48	184.15	4.07
S7.B.SF28	723.90	2.16	1	33.78	8.03	0.90	0.52	1.83	222.25	4.13
S7.C.SF28	720.85	2.15	1	32.51	8.17	0.86	0.56	1.64	225.55	4.22
S7.D.SF28	727.20	1.88	1	32.51	10.56	0.88	0.52	1.60	212.85	4.07

1 mm = 0.03937 inch.

**Table 5:** Statistical regression variables.

Equation	Coefficient of Multiple Determination R^2^, %	Standard Deviation S, mL/100 kg	Prob(t)						Prob (F)
			a	b	c	d	E	f	
1. HR_A_ = a + b*SF + c/b + d/b^2^	98.58	27.44	0.2847	0.0398	0.2897	0.2949	-	-	0.0211
2. VMA_A_ = a + b*SF + c/b + d/b^2^	99.53	22.61	0.0144	0.0187	0.0144	0.0146	-	-	0.0069
3. HR_B,C,D_ = a + b*SF + c/b + d/b^2^ + e/b^3^	92.68	27.16	0.0038	0.3983	0.0039	0.0039	0.0039	-	0.0000
4. VMA_B,C,D_ = a + b/SF + c*b + d/SF^2^ + e*b^2^ + f*b/SF	98.40	4.44	0.2027	0.0069	0.1913	0.00142	0.1809	0.0158	0.0000

**Table 6:** Influence of admixture sources on the fresh properties of SCCs.

Flowability/viscosity	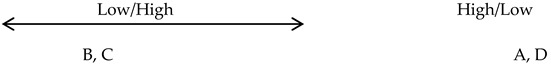
Dynamic stability	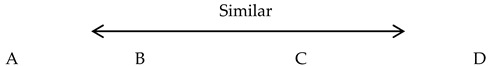
Static stability	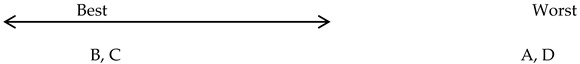
Passing Stability	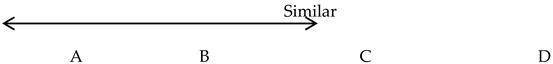
Filling Ability	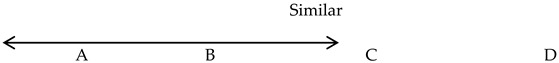

**Table 7:** Strength characteristics of studied self-consolidating concretes.

Mix No.	Demolded Unit Weight (kg/m^3^)	7 Days	28 Days	90 Days
S7.A.SF20	2452	44.21	55.50	69.24
S7.B.SF20	2452	45.13	56.68	70.85
S7.C.SF20	2452	45.67	57.42	71.62
S7.D.SF20	2452	44.79	56.22	70.16
S7.A.SF25	2452	44.72	56.32	70.13
S7.B.SF25	2452	45.58	57.41	71.64
S7.C.SF25	2452	46.13	57.92	72.48
S7.D.SF25	2452	45.39	56.99	71.04
S7.A.SF28	2452	45.55	57.08	70.43
S7.B.SF28	2452	46.41	58.30	71.98
S7.C.SF28	2452	47.02	59.03	72.87
S7.D.SF28	2452	46.19	57.86	71.31

## Data Availability

Data not available due to privacy and ethical restrictions.

## Conversion Factors

1 mm = 0.0394-inch	1 kg/m^3^ = 1.6856 lb/yd^3^
°C = (5/9) (°F-32)	1 mL/100 kg = 0.0153 oz/cwt
